# Electrical control of calcium oscillations in mesenchymal stem cells using microsecond pulsed electric fields

**DOI:** 10.1186/s13287-017-0536-z

**Published:** 2017-04-20

**Authors:** Hanna Hanna, Franck M. Andre, Lluis M. Mir

**Affiliations:** Vectorology and Anticancer Therapies, UMR 8203, CNRS, Univ. Paris-Sud, Gustave Roussy, Université Paris-Saclay, PR2, 114 rue Edouard Vaillant, 94805 Villejuif Cédex, France

**Keywords:** Mesenchymal stem cells, Calcium oscillations, Calcium spikes, Electroporation, Electric pulses, Electropermeabilization, Electropulsation

## Abstract

**Background:**

Human mesenchymal stem cells are promising tools for regenerative medicine due to their ability to differentiate into many cellular types such as osteocytes, chondrocytes and adipocytes amongst many other cell types. These cells present spontaneous calcium oscillations implicating calcium channels and pumps of the plasma membrane and the endoplasmic reticulum. These oscillations regulate many basic functions in the cell such as proliferation and differentiation. Therefore, the possibility to mimic or regulate these oscillations might be useful to regulate mesenchymal stem cells biological functions.

**Methods:**

One or several electric pulses of 100 μs were used to induce Ca^2+^ spikes caused by the penetration of Ca^2+^ from the extracellular medium, through the transiently electropermeabilized plasma membrane, in human adipose mesenchymal stem cells from several donors. Attached cells were preloaded with Fluo-4 AM and exposed to the electric pulse(s) under the fluorescence microscope. Viability was also checked.

**Results:**

According to the pulse(s) electric field amplitude, it is possible to generate a supplementary calcium spike with properties close to those of calcium spontaneous oscillations, or, on the contrary, to inhibit the spontaneous calcium oscillations for a very long time compared to the pulse duration. Through that inhibition of the oscillations, Ca^2+^ oscillations of desired amplitude and frequency could then be imposed on the cells using subsequent electric pulses. None of the pulses used here, even those with the highest amplitude, caused a loss of cell viability.

**Conclusions:**

An easy way to control Ca^2+^ oscillations in mesenchymal stem cells, through their cancellation or the addition of supplementary Ca^2+^ spikes, is reported here. Indeed, the direct link between the microsecond electric pulse(s) delivery and the occurrence/cancellation of cytosolic Ca^2+^ spikes allowed us to mimic and regulate the Ca^2+^ oscillations in these cells. Since microsecond electric pulse delivery constitutes a simple technology available in many laboratories, this new tool might be useful to further investigate the role of Ca^2+^ in human mesenchymal stem cells biological processes such as proliferation and differentiation.

## Background

Mesenchymal stem cells (MSCs) are multipotent stromal cells [1] originating from the embryonic mesoderm (mesenchyme) and present in many adult tissues such as bone marrow (bMSCs), adipose tissue (aMSCs), muscle, dermis, umbilical cord, etc. [2, 3]. These cells have gained a lot of momentum in the last decade due to their ability to differentiate into a wide variety of cells including osteoblasts, myoblasts, fibroblasts and chondrocytes. They also express key markers of cardiomyocytes, neuronal and endothelial cells [4]. This ability makes them a very promising candidate for cell therapy and regenerative medicine in order to heal damaged tissues and organs. However, MSCs from different tissues are not the same. They have different differentiation capacities and transcriptomic signatures [5].

Human-adipose MSCs (haMSCs), derived from adipose tissue are amongst the most easily accessible MSCs, with high quantities, and without aggressive extraction procedures. They are more available than other MSCs as, for example, the human bMSCs (hbMSCs). In addition, they have a phenotype, surface markers [6], and gene expression profile similar to those of the hbMSCs, and they are easier to maintain and proliferate [3], which make them ideal MSCs to use [7].

These cells present spontaneous Ca^2+^ oscillations, implicating Ca^2+^ channels and pumps of the plasma membrane (PM) and the endoplasmic reticulum (ER) [8]. These oscillations seem to start by an ATP autocrine/paracrine signaling [9] followed by inositol triphosphate (IP3)-induced Ca^2+^ release from the ER and further amplification from plasma membrane store-operated Ca^2+^ channels (SOCCs). Afterwards, the excess of Ca^2+^ is removed from the cytosol by the sarco/endoplasmic reticulum Ca^2+^ ATPase (SERCA), the plasma membrane Ca^2+^ ATPase (PMCA), and the Na^+^/Ca^2+^ exchanger (NCX) [10]. Ca^2+^ is one of the most important second messenger in the cell, and it regulates many important cellular processes such as ATP synthesis, apoptosis, cellular motility, growth, proliferation and gene expression. Hence, Ca^2+^ oscillations contain embedded information that has to be decoded by the cell, and Ca^2+^ signalling pathways play a key role in controlling cell behaviour and differentiation processes of MSCs.

It was shown that the Ca^2+^ oscillations frequency is different between undifferentiated MSCs and MSCs on route to differentiation and it differs between the various outcomes of the differentiation process (the level of differentiation and the differentiated cell type). While the MSCs exhibit regularly repeated Ca^2+^ oscillations, MSCs undergoing osteodifferentiation display a decrease in the frequency of their spontaneous Ca^2+^ oscillations while primary myoblasts present still another pattern of oscillations [11]. This shows that each cell type possesses its own pattern of Ca^2+^ oscillation frequency and shape, but the exact correlation between Ca^2+^ oscillations and MSCs differentiation is still unclear.

Presently, pulsed electric fields (PEFs) are widely used in research as a non-invasive physical means to permeabilize cellular membranes. Using one or several pulses of ultrashort duration causes changes in the cell membrane structure that permits access to the cell cytosol to molecules that cannot cross the plasma membrane under normal conditions [12]. Normally, Ca^2+^ is an ion that only crosses the plasma and ER membranes through channel proteins. Applying PEFs to cells in a medium containing Ca^2+^ (amongst other compounds) allows Ca^2+^ entry from the cell outside, and, if the electric field amplitude and pulse duration are high enough, Ca^2+^ is released from the cell inner stores [13]. The electromagnetic fields (EMFs) have been already applied on hbMSCs to test their effect on the differentiation of the latter. Those EMFs were either direct currents of very low electric field amplitude (0.1 V/cm) and a long time exposition (30 minutes) [14] or biphasic electric currents (1.5 μA/cm^2^ for 250 μs) [15]. These fields either activate voltage-operated Ca2+ (VOCCs) if their amplitude is large enough or do not alter the membrane potential if the electric field is too low. None of the electromagnetic fields used was permeabilizing the cell PM.

The novelty of our study is the use of PEFs of a high field amplitude that are capable of permeabilizing the cell PM or both the PM and the ER. Our objectives were to test the effect of these pulses on the Ca^2+^ oscillations of the cells. Attached haMSCs presenting normal spontaneous Ca^2+^ oscillations were exposed to one single pulse with a duration of 100 μs and different electric field amplitudes. At low and moderate electric field amplitudes, the Ca^2+^ spike induced by such PEFs can be very similar to a spontaneous oscillation. At high electric field amplitudes, the electrically induced Ca^2+^ spikes inhibit the spontaneous oscillations occurrence for some time after which oscillations reappear. This inhibition allowed us to impose on the cells experimentally induced Ca^2+^ spikes at a desired frequency. Hence, we show here that it is possible to control haMSCs spontaneous Ca^2+^ oscillations by microsecond pulsed electric fields (μsPEFs) with a complete preservation of the cell viability. The delivery of μsPEFs constitutes an easy way to control Ca^2+^ oscillations in mesenchymal stem cells, through their cancellation or the addition of supplementary Ca^2+^ spikes. Indeed, unlike chemical factors, which cause continuous effects, the direct link between the microsecond electric pulse(s) delivery and the ulterior occurrence/cancellation of the cytosolic Ca^2+^ spikes allowed us to mimic and regulate the Ca^2+^ oscillations in these cells. Since microsecond electric pulses delivery constitutes a simple technology available in many laboratories, the electrical control of Ca^2+^ oscillations could be in the future a promising tool to further investigate the role of Ca^2+^ in MSCs physiology and to better understand the correlation between the Ca^2+^ oscillations and MSCs biological processes such as proliferation and differentiation.

## Methods

### Cells and cell culture conditions

HaMSCs were isolated from surgical waste of individuals undergoing elective lipoaspiration. Samples were obtained after written informed consent from all the donors, in accordance with French and European legislations. The lipoaspirates were surgical waste and as such the French legislation (Art.L. 1245-2 du Code de la Santé Publique) establishes that the authorisation from an ethics committee is not required. Every experiment was done on cells before passage 8 from at least two donors (seven different donors in total). All haMSCs reacted similarly to the electric pulses, whoever the donor. The cells were grown in Dulbecco’s Modified Eagle Medium (DMEM) with Glutamax and supplemented with 10% fetal bovine serum, 100 U/mL penicillin and 100 mg/mL streptomycin and were cultured at 37 °C in a humidified incubator with 5% CO_2_. Cells were passed twice a week (every passage corresponds to one doubling time of the population). The multipotency capabilities of the cells were assessed by submitting them to differentiation conditions as previously reported in Liew et al. [16]. Cells from all the donors have thus been differentiated in osteoblasts, adipocytes and chondrocytes. The cell culture chemicals were purchased from Fischer Scientific (Parc d’innovation Illkirch, France).

### Cell staining

One day prior to the experiments, cells were seeded in 24-well plates at a density of 20 × 10^3^ cells/cm^2^. After 1 day, the cells were incubated for 30 minutes with 5 μM of Fluo-4 AM (Fischer Scientific), a fluorescent Ca^2+^ marker, in a humidified 5% CO_2_ atmosphere at 37 °C in complete DMEM. The incubation buffer also contained 375 nM of the nuclear fluorescent dye Hoechst 33342. After incubation, the attached cells were rinsed three times with PBS (phosphate-buffered saline) and 500 μl of complete DMEM were added to the cells.

### Microsecond pulse generator and electrodes

One single micropulse of 100 μs was delivered in all the experiments. A Cliniporator™ (Igea, Carpi, Italy) was used for the generation of the microsecond pulsed electric fields (μsPEFs). In order to treat the cells under the microscope, the pulse generator was connected to two parallel stainless steel rods of 1.2 mm diameter used as electrodes. The distance between them was 5 mm, and they were shaped to enter a 24-plate well and to reach the bottom of the dish. The whole system was set under a Zeiss Axiovert S100 epifluorescence inverted microscope.

In order to test the cell viability, another model of electrodes was designed. In this system, a thick cover of 10 cm^2^ containing two slots was designed to fit into a Petri dish of the same dimensions. Two plate electrodes of 2 mm thickness and 2 cm length were slipped into the slots until they touched the bottom of the Petri dish. This system does not permit observation of the cells under a microscope, it was used because it allows covering a larger surface (in order to have enough cells) than the system described above.

### Images acquisition and analyses

Images of the cells were taken every 10 seconds for 10 to 20 minutes with a Zeiss AxioCam Hrc camera controlled by the Axio Vision 4.6 software (Carl Zeiss, Oberkochen, Germany). The electric pulse was delivered after at least 2 minutes of recording, and 2 seconds before the next image. The excitation and emission wavelengths used for Fluo-4 were 496 nm and 515 nm respectively. The nuclear dye Hoechst 33342 (λex = 350 nm, λem = 461 nm) was used to recognise the nuclei and to track the cells using the Cell Profiler (version 2.0) software (Broad Institute, Cambridge, MA, USA). The software allowed the automatic measurement of the fluorescence intensity signal of each cell on every image. Curves were plotted using a Matlab program (version 7.8.0, The MathWorks Inc., Natick, MA, USA). All the observations were done at room temperature. The minimum opening time of the shutter for the fluorescent light was about 500 ms. To decrease the light energy applied on the cells, a 90% density Filter NE110B (Thorlabs, Maisons-Lafitte, France) was used.

### Cell viability assessment

One day prior to the experiments, cells were seeded at a density of 20 × 10^3^ cells/cm^2^ in 10-cm^2^ Petri dishes containing a 12 × 32 mm cover slide. After 24 hours, medium was removed, cells were washed with PBS, and 1 ml of fresh medium was added. The electrodes were placed on the cover slide using the Petri dish cover containing two slots and a 100-μs pulse was delivered to the attached cells. After the pulse, the Petri dish was put in the incubator for 20 minutes. Then, the non-pulsed cells on the cover slides were scratched under a sterile hood. The area covered by the pulsed cells was recognised due to the loss of cells beneath the electrodes after the removal of the latter. The cover slide with the pulsed cells only was transferred to a Petri dish with fresh medium and placed for 2 hours in the incubator. Then medium was removed and the cells were trypsinised, centrifuged, and resuspended in 300 μl of a fresh medium. This volume was distributed in two wells of an opaque-walled 96-multiwell plate and put in the incubator for 24 hours. After 24 hours, cells were visualised on an epifluorescence microscope to compare their shape and level of confluence, and 150 μl of Cell-Titer Glo Reagent (Promega, Madison, WI, USA) were added to each well according to manufacturer’s protocol. The reagent caused cell lysis and the generation of a luminescent signal proportional to the amount of the ATP present in the medium. A GloMax luminometer (Promega) was used to read the luminescence. The ATP amount and therefore the luminescence intensity reflected the number of cells present in the culture [17].

### Statistical analysis

All the experiments were done at least three times. Data are presented as means and standard deviations. The Spearman correlation was used to analyse the data of Figs. 3 and 6.

## Results

### Spontaneous Ca^2+^ oscillations in haMSCs in normal conditions and after one 100-μs electric pulse

**Fig. 1:**
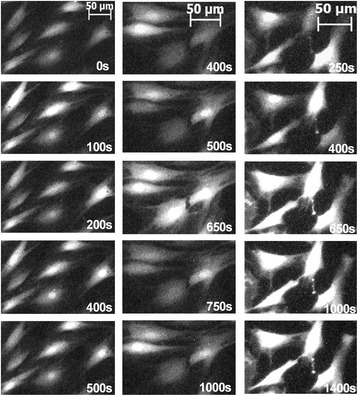
Time-lapse follow-up of calcium oscillations and spikes in haMSCs. *Left*: spontaneous calcium oscillations. *Middle*: spontaneous calcium oscillations before and after a 100-μs pulse of 300 V/cm (the pulse was applied at 630 s and a calcium spike in all the cells follows the pulse delivery at e.g. 650 s). *Right*: spontaneous calcium oscillations before a 100-μs pulse of 900 V/cm (applied at t = 630 s) followed by a non-oscillating increased calcium signal

Undifferentiated haMSCs present asynchronous Ca^2+^ oscillations as seen in Fig. 1 (left column). The oscillation frequencies differed from cell to cell and not all of the cells displayed spontaneous Ca^2+^ oscillations. When one electric pulse of 100 μs was applied on haMSCs, different situations could be observed. As shown in Fig. 1 (middle column), an electric pulse of 300 V/cm induced a Ca^2+^ spike in all the cells whatever the stage of their Ca^2+^ oscillation at the time the pulse was applied. After the pulse, the spontaneous Ca^2+^ oscillations returned to their original cell-dependent frequency and continued normally. When the electric pulse applied was 900 V/cm (Fig. 1, right), all the cells presented a Ca^2+^ spike with higher amplitude with respect to 300 V/cm. However, even 750 s after the pulse, the cells still presented a high cytosolic Ca^2+^ concentration (as shown by the persistent Fluo-4 high fluorescence level) and the spontaneous Ca^2+^ oscillations did not yet resume.

### Ca^2+^ spikes in response to different electric field amplitudes

**Fig. 2:**
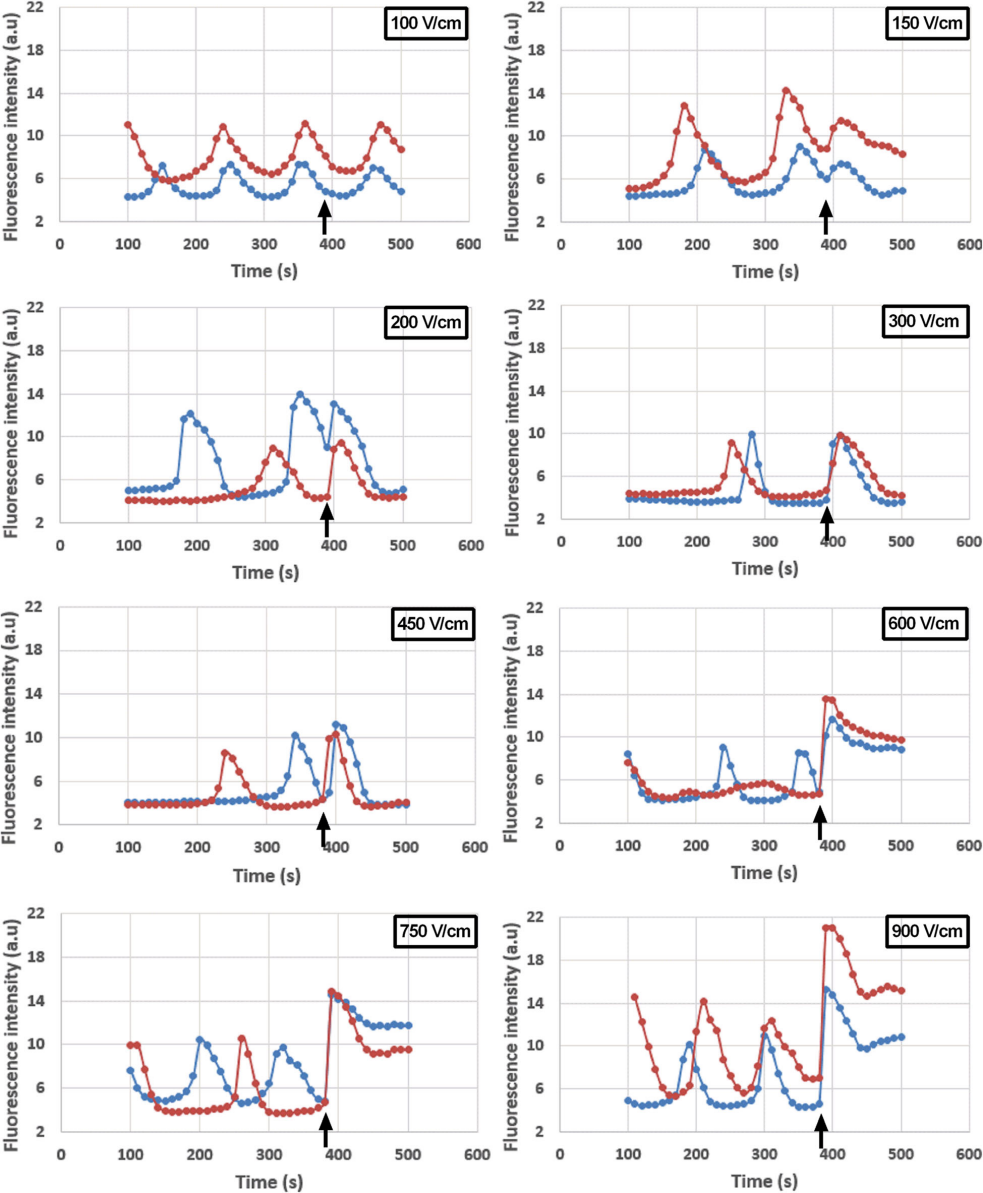
Calcium spikes in response to different electric field amplitudes. For each panel, two separate adherent cells, displaying the typical behaviour of the cells after the delivery of one 100-μs electric pulse, were chosen to illustrate that behaviour. In all the cases, the pulse was applied at time = 390 s (*black arrows*)

The shapes of the electrically induced Ca^2+^ spikes in response to a 100-μs duration pulse of different electric field amplitudes are represented in Fig. 2. At a very low electric field amplitude (100 V/cm), no Ca^2+^ spike was observed. At 150 V/cm, the Ca^2+^ spikes had a lower amplitude than the spontaneous oscillations and presented a relatively small slope. At moderate electric field amplitudes (200 to 450 V/cm), each electric pulse induced a Ca^2+^ spike. The Ca^2+^ spike displayed a higher slope than at 150 V/cm, then reached its maximal amplitude and came back to the normal Ca^2+^ amplitude as before the pulse. The electro-induced Ca^2+^ spikes had nearly the same shape and amplitude as the spontaneous oscillations at 200 and 300 V/cm. At 450 V/cm, their amplitudes were higher than the spontaneous ones, but they came back to the original baseline. When high electric field amplitudes were applied (600 to 900 V/cm), the electrically induced Ca^2+^ spike presented larger slopes (with different degree of sharpness depending on the electric field strength) and displayed a higher amplitude than the spontaneous oscillations; after the Ca^2+^ spike, the Ca^2+^ amplitude remained relatively high compared to the initial amplitude, and it took a longer time (several minutes) to come back to normal depending also on the amplitude of the applied electric field.

**Fig. 3:**
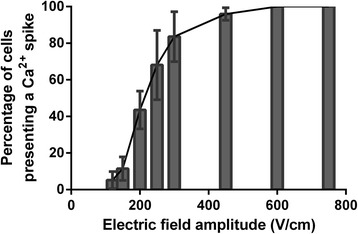
Percentage of cells presenting a calcium spike in response to one 100-μs electric pulse as a function of the field amplitude. r = 0.96 according to Spearman correlation

According to Fig. 3, only 10% of the cells presented a Ca^2+^ spike in response to the electric pulse at 150 V/cm. This percentage increased proportionally to the electric field amplitude, mainly between 150 and 300 V/cm. One hundred percent of the cells responded to the pulse at 450 V/cm. According to Spearman correlation analysis, there was a perfect correlation between the electric field amplitude and the percentage of cells displaying a Ca^2+^ spike (r = 0.96).

### Different outcomes on Ca^2+^ oscillations according to the electric field amplitude

**Fig. 4:**
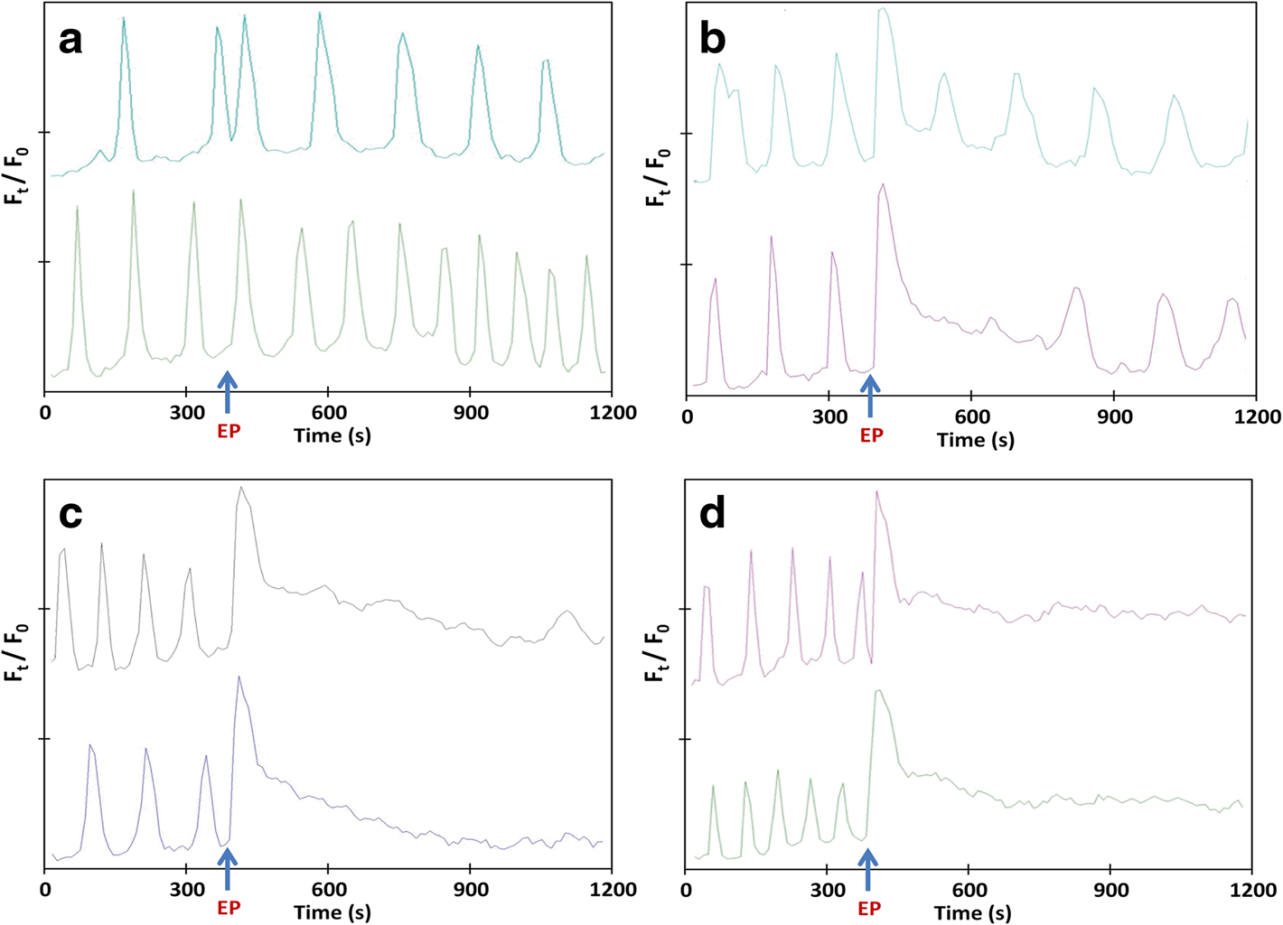
Examples of changes in the pattern of Ca^2+^ oscillations caused by the delivery of one 100-μs electric pulse of different field amplitudes, **a** 300 V/cm, **b** and **c** 600 V/cm, **d** 900 V/cm

According to the electric field amplitude, the pulse-induced Ca^2+^ spike had different effects on Ca^2+^ oscillations. At a relatively low electric field amplitude (300 V/cm), the pulse-induced Ca^2+^ spike displayed a very similar shape to the spontaneous Ca^2+^ oscillations (Fig. 4a), and it could be generated between two oscillations or during an oscillation. After the 100-μs pulse-induced Ca^2+^ spike, the spontaneous oscillations continued normally at their proper initial rhythm with no significant modification of the amplitude or duration of the oscillations. At relatively high electric field amplitude such as 600 V/cm, the pulse-induced Ca^2+^ spike displayed higher amplitude than the spontaneous oscillations and three different outcomes could be observed after the electro-induced spike. The oscillations could continue normally, with a minor change of the oscillation shape (Fig. 4b, upper trace). Or, second, a short inhibition of the spontaneous oscillations could be observed for some minutes, after which the oscillations reappeared (Fig. 4b, lower trace). The first oscillations that appeared after such an inhibition period did not display the same shape as the original ones. The third possible outcome was a longer inhibition of the Ca^2+^ oscillations, for a duration of longer than 10 minutes (Fig. 4c). The application of higher electric field amplitude (750 and 900 V/cm) resulted in the inhibition of the spontaneous Ca^2+^ oscillations for longer times. For example, only some cells had recovered their oscillations 45 to 60 minutes after the pulse delivery. However, after 24 hours all cells recovered their oscillations whatever the electric field applied (data not shown).

**Fig. 5:**
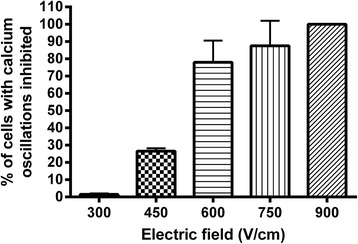
Percentage of cells displaying no Ca^2+^ oscillation within a period of at least 10 minutes after the delivery of one 100-μs electric pulse of different field amplitudes

**Fig. 6:**
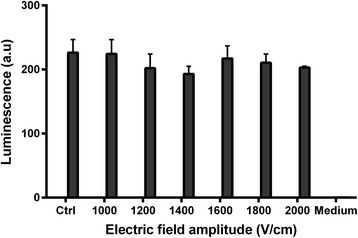
haMSCs cell viability after the delivery of one 100-μs electric pulse of different field amplitudes, r = 0.43 according to Spearman correlation

The percentage of cells with no spontaneous oscillation for at least 10 minutes after the electric pulse delivery increased as a function of the electric field amplitude (Fig. 5). While no inhibition of the spontaneous oscillations was observed at 300 V/cm, 25% of the cells had their Ca^2+^ oscillations inhibited after one electric pulse at 450 V/cm and nearly all the cells at 750 V/cm. Twenty-four hours after the pulse delivery, the cells remained viable under all the conditions tested, even for the highest electric field amplitude (Fig. 6). Photos taken before the Cell-Titer Glo assay showed no difference in the shape and level of confluence of the cells between the pulsed and the control wells (data not shown). The Spearman correlation showed no effect of the electric field amplitude on the cell viability (r = 0.43).

### Electrically mediated Ca^2+^ spikes manipulation after the 900 V/cm inhibition

**Fig. 7:**
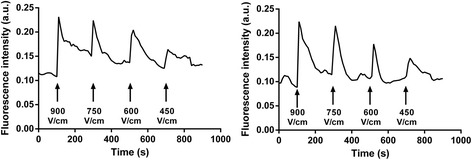
Two examples of calcium spike manipulation in haMSCs. After the delivery of one 100-μs electric pulse at 900 V/cm (that abolishes the spontaneous Ca^2+^ oscillations), pulses of various field amplitudes were delivered to mimic Ca^2+^ oscillations at experimentally controlled times

To test whether the cells were in a “refractory” state after the inhibition of their Ca^2+^ oscillations, or if they were still able to respond to the electric pulses, pulses of different field amplitudes were applied after a pulse of 900 V/cm (which provokes the absence of further spontaneous oscillations in 100% of the cells). For example (Fig. 7), a sequence of three pulses with descending electric field amplitudes (750 followed by 600 and 450 V/cm) was applied successively: the cells responded to the electric pulses as if each pulse would have been applied alone, or independently. The amplitude of the electrically induced Ca^2+^ spike was again proportional to the electric field amplitude like in Fig. 2. Thus calibrated electric pulses allow restoration of Ca^2+^ spikes at the time and with the amplitude chosen by the investigators.

## Discussion

Mesenchymal stem cells (MSCs) are multipotent stromal cells that have the ability to self-renew and to differentiate into a wide variety of cells of the mesodermal, ectodermal, and endodermal lineages [18]. This is why they represent an important tool for cell therapy and regenerative medicine. However, the differentiation procedures may be long and not able to respond to the demand. The improvement and optimisation of these procedures is of a crucial importance to ensure the differentiated cells in a reasonable time. Many studies have tested the effects of physical stimuli on the differentiation outcomes. Altman et al. showed that the application of a cyclic mechanical stimulation (translational and rotational strain) to undifferentiated MSCs (embedded in a collagen gel) over a period of 21 days resulted in ligament cell lineage differentiation even without differentiation factors [19]. Ward and colleagues studied the effect of the application of a 3–5% tensile strain to a collagen I substrate and concluded that it stimulated osteogenesis of attached hMSCs [20]. Low-intensity ultrasounds, another form of mechanical stress, have been shown to enhance the chondrogenic differentiation [21]. Sun et al. demonstrated that 0.1 V/cm electrical stimulation applied for 30 minutes per day for 10 days, on attached MSCs cultured with osteoinductive factors, accelerated their osteodifferentiation [14].

Ca^2+^ oscillations play an important role in the transduction of many physical stimuli in the cells. For example, tissue strain and shear stress on mouse tibia are transduced by repetitive Ca^2+^ spikes in the osteocytes. The spikes frequency and amplitude depend on the mechanical magnitude [22]. Electromagnetic fields (EMFs) can also promote MSCs differentiation to osteoblasts through Ca^2+^-related mechanisms. The exposition of MSCs to pulsed EMFs for 10 minutes every day results in the enhancement of osteogenesis early stages [23].

Ca^2+^ oscillations are a universal mode of Ca^2+^ signalling in both excitable [24, 25], and non-excitable cells [26]. In non-excitable cells such as hMSCs, Ca^2+^ oscillations are typically initiated by a receptor-triggered production of IP3 (after the binding of an agonist to the receptor) [27] and the subsequent Ca^2+^ release from the ER [28], which stimulates the store-operated calcium entry (SOCE) via the SOCCs [29]. It was shown that the Ca^2+^ oscillation frequency is different between undifferentiated MSCs and MSCs on route to differentiation and it differs between the various differentiated cell types [11]. According to Titushkin et al., the MSCs Ca^2+^ oscillations are suppressed after the addition of neuroinductive factors, but reappear in less than 7 days after neurodifferentiation [11]. Sun and colleagues observed that multipotent MSCs present 8.06 ± 2.64 Ca^2+^ spikes per 30 minutes of observation, and that this number decreases to 3.66 ± 2.42 after 28 days of incubation with osteoinductive factors [14].

In our study, we decided to analyse the effect of a type of short electric pulses termed microsecond pulsed electric fields (μsPEFs) on the spontaneous Ca^2+^ oscillations of haMSCs because many of the physical stimuli are transduced by cytosolic Ca^2+^ concentration rapid changes (oscillations) in the cells. The ultrashort pulses (one thousandth to one ten thousandth shorter than the pulses of 100 μs used in our study) called nanopulses or nanosecond pulsed electric fields (nsPEFs) have been used already to induce Ca^2+^ bursts in the cell, which resulted from the electropermeabilization of the ER, the plasma membrane or both [13, 30]. However, contrary to the 100-μs electric pulses, the technology to deliver nanopulses is not yet spread in the laboratories and is not simple to use.

The haMSCs loaded with the fluorescent marker Fluo-4 asynchronously displayed periodic changes in their cytosolic Ca^2+^ concentration detected by the periodic increase of their fluorescence. The videos of the haMSCs showed that not all the cells presented Ca^2+^ oscillations at a specific time. Some of the cells never displayed oscillations during the recording time (20 minutes). This observation can be related to the fact that MSCs display Ca^2+^ oscillations only during the phases G1 and S of the cell cycle, and not all the cells in the visualisation area of an experiment were in those phases of the cell cycle. Ca^2+^ oscillations increase the levels of cell cycle regulators such as cyclins A and E and probably control cell cycle progression and cell proliferation, via the regulation of cyclin levels (amongst other mechanisms) [31]. In addition, the haMSCs presenting Ca^2+^ oscillations displayed them at different rhythms and frequencies: the oscillations were asynchronous, and at a given time, each cell was in a different phase of a Ca^2+^ oscillation and the oscillations frequencies displayed wide variations between cells. The average duration of an oscillation was around 2 minutes.

Electric pulses of 100 μs duration are already applied in many biotechnological and medical applications, notably in anticancer electrochemotherapy [32], tumour ablation [33], and cell transfection [34]: by permeabilizing the plasma membrane temporally they allow the internalisation of non-permeant molecules of interest like drugs or nucleic acids. Classically, eight successive pulses of 100 μs are used in biomedical sciences. The technology is spread in many laboratories and is very simple to use. In our study, we applied a single electric pulse because, when several pulses are delivered, pulses repetition frequency may impact the efficacy of cell membrane permeabilization as we have recently shown in our group [35]. When an electric pulse of 100 μs was applied to the cells, an electrically induced Ca^2+^ spike was observed synchronously in a given percentage of the cells (as a function of the electric field amplitude). Moreover, the electric field amplitude should be enough to cause the transmembrane voltage to reach a permeabilization threshold. This threshold depends on the size of the molecule to be internalised. This is why, at 100 V/cm, which is a very low electric field amplitude, no Ca^2+^ spike was observed because there was no permeabilization and hence no Ca^2+^ entry. 120 V/cm was the lowest electric field amplitude at which some cells presented Ca^2+^ spikes. This threshold is very low compared to other ones previously reported, because the Ca^2+^ has a small size compared to other classical markers such as yo-pro-1 iodide, propidium iodide or bleomycin that are those frequently used (Hanna et al., submitted). At 450 V/cm, 100% of the cells responded to the electric pulse. The origin of the Ca^2+^ causing the Ca^2+^ spike was not always the same: it could be the result of Ca^2+^ entry from the external medium only, or the combination of Ca^2+^ entry from cells outside and Ca^2+^ release from the ER. Our group demonstrated recently that the μsPEFs could permeabilize not only the cell membrane (as usually observed and stated) but also the internal organelles membranes (Hanna et al., submitted). The origin of the Ca^2+^ spike depends on the electric field amplitude. If the latter is below 500 V/cm, the pulse permeabilizes only the plasma membrane (Hanna et al., submitted), and the Ca^2+^ spike is primarily the result of Ca^2+^ entry through the plasma membrane followed by an amplification due to Ca^2+^ entry from the voltage-operated Ca^2+^ channels (VOCCs), activated by the membrane depolarisation [36]. However, above 500 V/cm, the electric pulses will also affect the ER membranes, permeabilize them and cause Ca^2+^ release from the ER (Hanna et al., submitted). Hence, the Ca^2+^ spike at high electric field amplitudes, resulted primarily of a massive Ca^2+^ entry through the plasma membrane (due to the generation of larger pores at the higher field amplitudes) also followed by the external Ca^2+^ entry through the VOCCs, the Ca^2+^ release from the ER and the subsequent stimulation of the store-operated Ca^2+^ channels (SOCCs) [37]. This explains why the Ca^2+^ spike induced by electric fields below 500 V/cm had a very similar shape to the spontaneous Ca^2+^ oscillations with a gradual increase and same amplitude, whereas the Ca^2+^ spike induced by electric fields above 500 V/cm had higher amplitude than the spontaneous oscillations, and presented a sharper rise (due to the abrupt penetration of Ca^2+^ through the permeabilized membranes). At 600 V/cm, three different outcomes were observed, mainly due to four factors: first the distribution of the electric field which results in field amplitude small differences in the area between the two electrodes; second, the cells are heterogeneous in size and according to Schwan equation [38], the electric field impact will be different between the smallest and the largest cells; third, the orientation of the cell with respect to the electric field lines, which has an impact on the induced transmembrane potential that will lead to the cell membrane permeabilization [39]; fourth, the position of the cells are in the cell cycle that could lead to different responses to the pulses. However, at 750 V/cm, it seems that the electric field has exceeded a certain threshold above which no more heterogeneity was observed and nearly all the cells had their Ca^2+^ oscillations inhibited.

The Ca^2+^ spikes obtained looked like the Ca^2+^ spikes induced by even shorter electric pulses (the so-called nanopulses, of a duration of e.g. 10 ns, that can directly affect the cell internal membranes such as those of the endoplasmic reticulum [30]). In our study, an electrically induced Ca^2+^ spike with properties very near to the oscillations did not inhibit the latter, could be applied at any time of an oscillation without interfering with the latter, and after the pulse, the oscillations continued normally at their own rhythm, and with their normal shape. Nevertheless, a high-amplitude Ca^2+^ spike inhibited the Ca^2+^ oscillations. The percentage of cells that were unable to display further Ca^2+^ oscillations increased proportionally with the electric field amplitude of the inhibitory electric pulse. The Ca^2+^ oscillations inhibition could last some minutes or be longer, to reach tens of minutes, also depending on the electric field amplitude of the inhibitory electric pulse.

The inhibition of the Ca^2+^ oscillations for some time allowed us to study the state of the cell during this period, to determine whether the cell was in a “refractory state” or if it was still able to respond to an electric pulse. The application of several pulses after a 900 V/cm pulse inhibition showed that the cells could still react to the pulse and present Ca^2+^ spikes with different amplitudes depending on the applied electric field amplitude.

Last but not least, the application of one 100-μs electric pulse did not affect the cell viability 24 hours after treatment (Fig. 6), even when applying a much stronger electric field than the one used to inhibit the calcium oscillations for several minutes. Moreover, in a previous study [16] we showed that haMSCs maintain their stem cell phenotype after being exposed to even stronger electric field conditions (eight electric pulses of 100 μs at 1500 V/cm and at a 1Hz frequency). More precisely:

1. Seven days after exposure, MSCs showed similar stem cell surface antigens expression compared with non-treated MSCs.
2. Four days after exposure to the eight electric pulses, MSCs were cultured in osteogenic and adipogenic differentiation media and successfully differentiated into osteoblasts and adipocytes.
3. Eleven days after exposure to the electric pulses, MSCs cultures yielded similar cell growth rates compared with non-treated MSCs cultures.

The electrically induced Ca^2+^ spikes can have wide effects on the Ca^2+^ oscillations frequency, amplitude and duration. Whereas the nanosecond pulses allowed only to add a Ca^2+^ spike to the oscillations [30], the microsecond pulses display more complex effects. The electric field amplitude allowed us either to induce a Ca^2+^ spike without affecting the oscillations, or to inhibit the Ca^2+^ oscillations and impose on the cell Ca^2+^ oscillations at a desired frequency and amplitude. It is worth noting that the microsecond technology is cheaper than the nanosecond one, it is more available in the laboratories, and simple generators can deliver it. These considerations add further value to the observations reported here. Taking into account that each type of differentiated cell from MSCs has its own Ca^2+^ oscillation characteristics, and that these oscillations may play an important role in the decision of MSCs fate, the μsPEFs constitute a novel useful tool to control the oscillations and the differentiation of MSCs.

## Conclusions

hMSCs show great promise for regenerative medicine. As a function of the pulse(s) electric field amplitude, we report here that it is possible to generate in hMSCs a supplementary calcium spike with properties close to those of their spontaneous calcium oscillations, or, on the contrary, to inhibit their spontaneous calcium oscillations for a very long time compared to the pulse duration. Through that inhibition of the oscillations, Ca^2+^ oscillations of desired amplitude and frequency could then be imposed on the cells using subsequent electric pulses. The delivery of short PEFs of 100 μs, a robust technology easy to apply, could be therefore a promising way to investigate the role of the Ca^2+^ in MSCs physiology and biological processes such as proliferation and differentiation.

## Abbreviations

aMSCs: Adipose tissue mesenchymal stem cells
bMSCs: Bone marrow mesenchymal stem cells
DMEM: Dulbecco’s Modified Eagle Medium
EMFs: Electromagnetic fields
ER: Endoplasmic reticulum
haMSCs: Human aMSC
hbMSCs: Human bMSC
IP3: Inositol triphosphate
MSCs: Mesenchymal stem cells
NCX: Na+/Ca2+ exchanger
PBS: Phosphate-buffered saline
PEFs: Pulsed electric fields
PM: Plasma membrane
PMCA: Plasma membrane Ca2+ ATPase
SERCA: Sarco/endoplasmic reticulum Ca2+ ATPase
SOCCs: Store-operated Ca2+ channels
VOCCs: Voltage-operated Ca2+ channels
μsPEFs: Microsecond pulsed electric fields
